# Isoliquiritigenin Prevents the Development of Nephropathy by an HFD in Rats Through the Induction of Antioxidant Production and Inhibition of the MD-2/TLR4/NF-κB Pathway

**DOI:** 10.3390/biology13120984

**Published:** 2024-11-28

**Authors:** Mohammed Abdo Yahya, Ghedeir M. Alshammari, Magdi A. Osman, Laila Naif Al-Harbi, Setah Naif Alotaibi

**Affiliations:** Department of Food Science and Nutrition, College of Food and Agricultural Sciences, King Saud University, Riyadh 11451, Saudi Arabia; mabdo@ksu.edu.sa (M.A.Y.); magdios@ksu.edu.sa (M.A.O.); lalharbi1@ksu.edu.sa (L.N.A.-H.); 443203416@student.ksu.edu.sa (S.N.A.)

**Keywords:** isoliquiritigenin, nephroprotection, high-fat diet, oxidative stress, inflammation, rats

## Abstract

This study tested the ISL against renal damage induced by a high-fat diet (HFD) and explored its underlying mechanisms. Adult male rats were assigned to four groups: (1) control on a standard diet (STD), (2) ISL on STD (30 mg/kg), (3) HFD, and (4) HFD + ISL (30 mg/kg). After 12 weeks of dietary intervention, ISL treatment led to significant reductions in body weight gain, visceral fat, and glucose and insulin levels in HFD-fed rats. Notably, ISL decreased serum urea and creatinine, increased serum albumin, and improved urinary profiles by lowering the urinary albumin and the albumin/creatinine ratio. Histological analyses revealed that ISL enhanced the glomerular structure and mitigated tubular damage, as evidenced by reduced urinary excretion of the kidney injury markers NGAL and KIM-1. Additionally, ISL significantly lowered cholesterol, triglycerides, and free fatty acids in both the control and HFD groups while also decreasing oxidized low-density lipoproteins (ox-LDLs) and malondialdehyde (MDA). Importantly, ISL enhanced renal antioxidant levels, increasing glutathione (GSH), superoxide dismutase (SOD), and catalase (CAT). Moreover, ISL downregulated mRNA levels of MD-2, Toll-like receptor-4 (TLR-4), and NF-κB, leading to reduced NF-κB p65 levels in renal tissues. In conclusion, ISL offers substantial protection against HFD-induced renal toxicity through mechanisms that attenuate metabolic stress, enhance antioxidant defenses, and inhibit the MD-2/TLR4/NF-κB inflammatory pathway.

## Simple Summary

Evidence suggests that inflammation mediated by the activation of various inflammatory signaling pathways contributes significantly to HFD-related renal damage. This study investigated the effects of the ISL against renal damage induced by a high-fat diet (HFD) and explored its underlying mechanisms. Generally, ISL enhanced renal antioxidant levels, increasing GSH, SOD, and CAT. Moreover, ISL downregulated mRNA levels of MD-2, Toll-like receptor-4 (TLR-4), and NF-κB, leading to reduced NF-κB p65 levels in renal tissues. Therefore, this study can provide a reference for the study of HFD-induced renal toxicity.

## 1. Introduction

Chronic kidney disease (CKD) remains a significant global health concern characterized by high mortality and morbidity rates [1]. Current estimates suggest that CKD affects approximately 9.1% of the population, leading to around 1.2 million deaths annually [2]. The progression of CKD can result from various damaging factors, including oxidative stress and inflammation, particularly following renal tubular injuries associated with conditions such as hyperglycemia, ischemia, and glomerulonephritis. These conditions are often marked by diminished renal function, persistent glomerulosclerosis, and interstitial fibrosis [3,4]. Ultimately, CKD can advance to end-stage renal disease (ESRD), necessitating dialysis or transplantation, and can contribute to other chronic complications including anemia, bone disease, and cardiovascular disorders [5,6].

Recent research has extensively explored the rising prevalence of CKD and effective management strategies [7,8]. A major focus has been on dietary patterns, particularly the shift toward Western-style diets, which are high in unhealthy fats, including saturated and trans fatty acids [9,10]. Numerous studies have linked these dietary patterns to increased CKD incidence [10,11,12,13], while healthier diets correlate with lower CKD prevalence [13,14]. Additionally, high-fat diets (HFDs) contribute significantly to CKD, primarily due to increased risk factors such as obesity and metabolic syndrome, which include hyperglycemia, dyslipidemia, hypertension, insulin resistance, and type 2 diabetes mellitus [10,15]. However, the exact molecular mechanisms through which the HFD induces nephropathy are not fully understood.

Evidence suggests that inflammation mediated by the activation of various inflammatory signaling pathways contributes significantly to HFD-related renal damage by promoting oxidative stress, fibrosis, and apoptosis [3,4,8,16]. In general, inflammation is central to the pathogenesis of renal diseases [17]. The nuclear factor kappa-light-chain-enhancer of activated B cells (NF-κB) is a key transcription factor that promotes chronic inflammation and oxidative stress by upregulating mediators like tumor necrosis factor α, IL-6, and IL-1β [18]. NF-κB is well documented in various renal disorders [18,19], and its activation is essential for renal damage in metabolic syndrome, diabetes, and HFD exposure, with pharmacological inhibition offering protective benefits [20,21,22,23,24,25]. However, among many inflammatory pathways, the Toll-like receptors (TLRs) were shown to have a crucial role in renal injury via NF-κB activation, requiring myeloid differentiation protein-2 (MD-2) to activate TLR4 [26,27,28,29]. Recent studies have identified the MD-2/TLR4/NF-κB axis as a key mechanism in HFD- and obesity-related renal damage, suggesting that targeting this pathway may provide a novel approach to mitigating HFD-induced nephropathy [8,30,31,32,33].

Isoliquiritigenin (ISL), (E)-1-(2,4-Dihydroxyphenyl)-3-(4-hydroxyphenyl)-2-propen-1-one, 4,2′,4′-Trihydroxychalcone, is a well-characterized active compound derived from the roots of Glycyrrhiza uralensis, commonly known as the licorice plant [34]. ISL exhibits diverse pharmacological properties in animal models, demonstrating anti-cancer, cardioprotective, hepatic, neuroprotective, and renal-protective effects, largely attributed to its antioxidant and anti-inflammatory capabilities through the modulation of pathways such as sirtuin-1 (SIRT1), nuclear factor erythroid factor-2 (Nrf2), and NF-κB [35,36,37]. Additionally, ISL has shown the ability to mitigate inflammation both in vitro and in vivo by inhibiting NF-κB signaling [38,39,40,41,42]. Previous findings from our research indicated that ISL protects against HFD-induced intestinal and hepatic damage through hypoglycemic and anti-hyperlipidemic actions as well as by suppressing inflammation via NF-κB inhibition [43,44]. Other studies have reported ISL’s nephroprotective effects against lipopolysaccharide-induced acute kidney injury and unilateral ureteral obstruction-induced renal damage, also mediated through NF-κB suppression [45,46].

Despite the extensive body of research, the specific role of ISL in countering HFD-induced renal damage and nephropathy has not yet been thoroughly investigated. Based on the available evidence, this study hypothesizes that chronic administration of ISL to HFD-fed rats could mitigate the nephropathy associated with high-fat diets. Furthermore, we aim to evaluate whether this protective effect is mediated through the modulation of the MD2/TLR4/NF-κB axis or through alternative protective mechanisms.

## 2. Materials and Methods

### 2.1. Animals

A cohort of thirty-two adult male Wistar-type rats, each weighing approximately 180 ± 20 g, was procured from the Experimental Animal Care Center at King Saud University (KSU) in Riyadh, Saudi Arabia. Throughout the study, the rats were housed in a dedicated environment designed to minimize stress, with consistent ambient conditions maintained at 22 ± 1 °C and a controlled 12 h light/dark cycle. These conditions were crucial, as they helped mitigate stress-induced factors that could influence experimental outcomes. All procedures involving the animals—ranging from handling and treatment to surgical interventions—were rigorously reviewed and approved by the Research Ethics Committee (REC) at KSU (Ethical Reference No: KSU-SE-21-34).

### 2.2. Diets and Drugs

The standard control diet (cat D12450B/10% fat/3.85 kcal/g) and HFD (cat D12451, comprising 45.6% kcal from fat) were procured from Research Diets, New Brunswick, NJ, USA. The detailed compositions of these diets have been previously elucidated in our studies [43,44]. Isoliquiritigenin (ISL, cat HY-N0102) was sourced as a pure powder from Med Chem Express, also based in New Jersey. To ensure optimal efficacy, ISL was freshly prepared in a 0.5% carboxymethylcellulose (CMC) vehicle, achieving the precise final concentration employed in our experiments.

### 2.3. Experimental Design

Thirty-two male Wistar-type rats were meticulously randomized into four distinct groups, with each group comprising eight rats to ensure robust statistical analysis and reliability of results. The groups were as follows: (I) Control group, which received the standard control diet along with a daily oral administration of 0.5 mL of 0.5% carboxymethylcellulose (CMC) as the vehicle (30 mg/kg) for a duration of 12 weeks; (II) ISL-treated group, which was also fed the standard diet but received daily oral doses of ISL, prepared in 0.5% CMC (0.5 mL/30 mg/kg), over the same 12-week period; (III) HFD-model group, which was subjected to a high-fat diet while receiving 0.5 mL of 0.5% CMC daily for 12 weeks; and (IV) HFD + ISL-treated group, which was administered the high-fat diet in conjunction with daily oral doses of ISL in 0.5% CMC (30 mg/kg) for the entire 12 weeks. To monitor the effects of the interventions, body weights were systematically recorded on a weekly basis, providing critical data on the metabolic impacts of the treatments. A 12-week chronic feeding of HFD as a model to induce diabetic nephropathy was previously described and validated by others [16]. Oral treatment with ISL (30 mg/kg) has been demonstrated in prior research to effectively suppress inflammation and oxidative stress by suppressing NF-κB in a rat model of NAFLD and unilateral ureteral obstruction (UUO) [43,44,47].

### 2.4. Urine, Plasma, Serum, and Tissue Collections

On the final day of the experimental protocol, urine samples were meticulously collected by housing each rat individually in metabolic cages. Following a fasting period of 12 h, the rats were anesthetized using a mixture of ketamine and xylazine hydrochloride (80/10 mg/kg, *w*/*w*). Blood was then drawn into Ethylenediaminetetraacetic acid (EDTA) and plain tubes to facilitate the collection of plasma and serum, respectively. Both urine and blood samples were subjected to centrifugation at 500× *g* for 15 min, with the resulting supernatants stored at −20 °C for subsequent analysis. Afterward, the rats were euthanized through cervical dislocation, and various adipose tissues—epididymal, retroperitoneal, inguinal, and peri-renal—were harvested, weighed, and preserved at −80 °C. The total inguinal fat was classified as subcutaneous fat (CF), while the combined mass of the remaining fat deposits was designated as visceral fat (VF) [16]. Concurrently, kidneys were carefully excised on ice and sectioned, and some fragments were fixed in 10% buffered formalin for histological examination. The remaining kidney tissues were snap-frozen in liquid nitrogen and stored at −80 °C until further use [48].

### 2.5. Biochemical Analysis in the Serum and Urine

Plasma glucose concentrations were determined using a colorimetric assay kit (Cat# CC-81695, CliniSciences, Nanterre, France). Plasma insulin levels were measured with an ELISA kit (cat ERINS, ThermoFisher, Waltham, MA, USA). Total cholesterol (CHOL) in serum and renal tissues was assessed using a rat-specific ELISA kit (cat 50-194-7653, Crystal Chem, Sacramento, CA, USA). LDL-c in serum was measured using an ELISA kit (cat# 79960, Crystal Chem, Sacramento, CA, USA). Triglyceride (TG) levels were evaluated with another rat-specific ELISA kit (cat NB-E30046, Novatein Biosciences, Woburn, MA, USA). Free fatty acids (FFAs) were quantified using an ELISA kit (cat E-BC-F039, Houston, TX, USA). Oxidized low-density lipoproteins (ox-LDL-c) in serum and renal samples were measured using an assay kit (cat NBP2-79692, Novus Biologicals, Centennial, CO, USA). Urea levels in both serum and urine were analyzed with a colorimetric kit (cat DIUR-100, BioAssay Systems, Hayward, CA, USA). Creatinine (Cr) and albumin (Alb) levels in serum and urine were quantified using enzyme-based kits (cat ab CSB-E14403r for Cr and cat CSB-E12991r for Alb, CUSABIO, Houston, TX, USA). Neutrophil gelatinase-associated lipocalin (NGAL) levels in urine were assessed using an ELISA kit (cat BPD-KIT-046, ENZO, Newark, TX, USA). Kidney injury molecule-1 (KIM-1) and 8-Hydroxy-2′-deoxyguanosine (8-OHdG) levels in urine were measured with specific ELISA kits (cat ab223858, Abcam, Cambridge, UK and MBS267513, myBioSource, San Diego, CA, USA, respectively). The creatinine excretion rate was calculated using the formula: [Cr excretion rate (mg/dL/day) = urinary Cr (mg/dL) × urine volume in 24 h], following established protocols [37,49]. All assays were performed in duplicate according to the manufacturer’s instructions, with a sample size of *n* = 8 per group.

### 2.6. Preparation of Kidney Homogenates

Tissue homogenates from frozen kidney samples were prepared with careful precision to ensure accuracy and consistency in the processing of specimens. Initially, 40 mg of frozen kidney tissue was weighed and kept on ice to prevent any degradation of proteins. A homogenization buffer was created by diluting phosphate-buffered saline (PBS) at a ratio of 1:10 (*w*/*v*) and supplemented with a protease and phosphatase inhibitor cocktail (ab271306, Abcam, Cambridge, UK) to achieve a final volume of 500 µL, thereby preserving protein stability. The tissue was finely minced and introduced into a pre-chilled homogenizer containing the buffer. Homogenization was performed on ice using a high-speed homogenizer set to 10,000 rpm for 30 s, ensuring a thorough and uniform suspension. Subsequently, the mixture was centrifuged at 1200× *g* for 10–15 min at 4 °C to eliminate cellular debris. The supernatant obtained was aliquoted into pre-chilled microcentrifuge tubes and stored at −80 °C for subsequent biochemical analysis.

### 2.7. Biochemical Analysis in the Renal Tissue Homogenate

Malondialdehyde (MDA), a key indicator of lipid peroxidation, was quantified in renal homogenates using a specific kit (cat. No. RTEB1739, AssayGenie, Dublin, DO, Ireland). Total glutathione (GSH) levels were assessed with another rat-specific kit (cat orb782371, MyBiosources, biorbyt, Durham, NC, USA), while superoxide dismutase (SOD) activity was measured using a dedicated kit (cat ARG81353, arigo, Zhubei, Hsinchu County, Taiwan). Catalase (CAT) levels were evaluated with a specific assay (cat MBS266404, MyBiosources, San Diego, CA, USA). Additionally, tumor necrosis factor-α (TNF-α) levels in the homogenates were analyzed using an ELISA kit (cat E-EL-R2856, Elabscience, Houston, TX, USA), and interleukin-6 (IL-6) levels were also measured via a specific ELISA kit (cat E-EL-R0015, Elabscience, Houston, TX, USA). All measurements were performed in duplicate and followed the manufacturer’s protocols, with each group consisting of 8 samples.

### 2.8. Isolation of the Cytoplasmic and Nuclear Fractions and Biochemical Measurements

For the isolation of nuclear proteins, the Active Motif Nuclear Extract Kit (40010, Active Motif, Tokyo, Japan) was used, and it utilized 50 mg of the tissue. In brief, each kidney sample was thawed and finely chopped to facilitate homogenization in 500 µL of ice-cold PBS, which was enhanced with 1 mM sodium orthovanadate and 1 mM sodium fluoride to maintain protein integrity. The resulting homogenate was subsequently mixed with 500 µL of a hypotonic lysis buffer composed of 10 mM HEPES (pH 7.9), 10 mM KCl, 1.5 mM MgCl_2_, and 0.5% NP-40. After a 15-min incubation on ice, centrifugation at 1000× *g* for 5 min at 4 °C was performed to separate the cytoplasmic and nuclear components. The supernatant, which contained the cytoplasmic proteins, was carefully collected and stored at −80 °C for future analysis. The nuclear pellet was resuspended in 100 µL of a detergent-free lysis buffer consisting of 20 mM HEPES (pH 7.9), 400 mM NaCl, and 1.5 mM MgCl_2_, along with a protease inhibitor cocktail. After a 30 min incubation on ice with periodic vortexing, the mixture was centrifuged at 14,000× *g* for 10 min at 4 °C, yielding a clear nuclear extract. Total protein content was determined by Bradford protein assay kit (Catalog ID 5000201EDU, BioRad, Feldkirchen, Germany). The purity of the cytoplasmic and nuclear fractions was confirmed in the cytoplasmic and nuclear fractions by measuring specific cytoplasmic and nuclear marker proteins, namely, GAPDH and Histone H3, by specific ELISA kits. The cytoplasmic and nuclear levels of NF-κB were determined through an ELISA kit (cat MBS2505513, MyBioSource, San Diego, CA, USA). All measurements were performed in duplicate and followed the manufacturer’s protocols, with each group consisting of 8 samples.

### 2.9. Real-Time Polymerase Chain Reaction (qPCR)

The primer sequences for amplifying TLR4, MD-2, and NF-κB were sourced from ThermoFisher (Carlsbad, CA, USA) and are listed in Table 1. RNA isolation was performed on a 20 mg kidney sample from each group using TRIZOL reagent (ThermoFisher, Carlsbad, CA, USA). Subsequently, cDNA was synthesized in the laboratory utilizing a dedicated cDNA synthesis kit (cat K1621, ThermoFisher, Carlsbad, CA, USA). Amplification reactions were executed in a CFX96 real-time PCR machine with the Ssofast Evergreen Supermix kit (cat 172-5200, BioRad, Hercules, CA, USA). The amplification protocol consisted of an initial heating step (1 cycle at 98 °C for 30 s), followed by 40 cycles of denaturation at 98 °C for 5 s and annealing/extension at 60 °C for 5 s. A melting curve analysis was conducted with a final cycle at 95 °C for 5 s. Each target gene expression was normalized to its corresponding reference gene using the 2^−ΔΔCT^ method to ensure accurate quantification. All analyses were performed in duplicate.

### 2.10. Histopathological Evaluation

For the histological assessment of kidney tissues, the procedure commenced with the rehydration of formalin-preserved samples through a sequential ethanol gradient. Initially, the tissues were immersed in 70% ethanol for 30 min, followed by transitions to 80%, 90%, and finally 100% ethanol, with each step lasting 30 min to ensure thorough dehydration. Following this, the samples were treated with xylene for 30 min to facilitate the removal of ethanol. After clearing, the tissues were infiltrated with paraffin wax at a maintained temperature of approximately 60 °C for a minimum of 2 h, ensuring complete embedding. Once embedded, the specimens were sectioned into 5 µm slices using a microtome and affixed onto glass slides. Routine staining was performed using hematoxylin and eosin (HE); sections were deparaffinized in xylene, followed by rehydration through a decreasing ethanol series. The tissues were stained with hematoxylin for 5–10 min, subjected to differentiation in hydrochloric acid, and counterstained with eosin. After another round of dehydration and clearing, the slides were mounted with coverslips for protection. The finalized stained sections were analyzed under a light microscope at a magnification of 200×, allowing for the capture of detailed images reflecting the tissue morphology and any pathological alterations.

### 2.11. Statistical Analysis

Data obtained from all measurements were tested for normality using the Kolmogorov–Smirnov test. Data were analyzed using GraphPad prism analysis software (v8, Boston, MA, USA), one-way ANOVA, and Tukey’s test as a post hoc test. The levels of significance were considered at *p* < 0.05.

## 3. Results

### 3.1. ISL Lowers Fat Deposition and Plasma and Glucose Levels in HFD-Fed Rats

No significant differences in body weight nor levels of visceral fats, subcutaneous fats, plasma glucose, or plasma insulin were seen between the control and ISL-treated rats (Table 2). The levels of all these parameters were significantly increased in the HFD-fed rats compared with the control rats and were significantly reversed in the HFD + ISL-treated rats compared with the HFD-fed rats (Table 2).

### 3.2. ISL Attenuates Dyslipidemia and Attenuates Lipid Accumulation in the Kidneys of HFD-Fed Rats

Significantly higher levels of total CHOL, TGs, ox-LDL-c, and FFAs were seen in the serum and kidney tissues of the HFD-fed rats compared with the control rats (Table 3). In addition, higher serum levels of LDL-c were seen in the HFD-fed rats compared with the control rats (Table 3). On the other hand, a significant reduction in the serum and renal levels of all lipids was observed in both the ISL-treated and HFD + ISL-treated rats compared with either the control or HFD-fed rats, respectively (Table 3).

### 3.3. ISL Improves Kidney Function in HFD-Fed Rats

Compared with the control group, the HFD-fed rats had a significant increase in kidney weight, and their serum showed significantly higher levels of urea but significantly lower albumin content (Table 4). The urine volume of the HFD-fed rats was also reduced compared with the control rats. In addition, urine samples of the HFD-fed rats showed significantly higher urinary levels of albumin, 8-OHdG, NGAL, and KIM-1; low levels of Cr; and higher ratios of albumin/Cr compared with the control rats (Table 4). While no variations in the levels of all these markers were seen between the control and ISL-treated rats, the levels of all these markers were significantly reversed in the HFD + ISL-treated rats compared with the HFD-model rats (Table 4). Among all these serum and urinary markers, only the levels of 8-OHdG were significantly lower in the ISL-treated rats than the control rats (Table 4).

### 3.4. ISL Boosts Antioxidant Levels, Inhibits Lipid Peroxidation, and Reduces Markers of Inflammation in the Kidneys of HFD-Fed Rats

**Figure 1:**
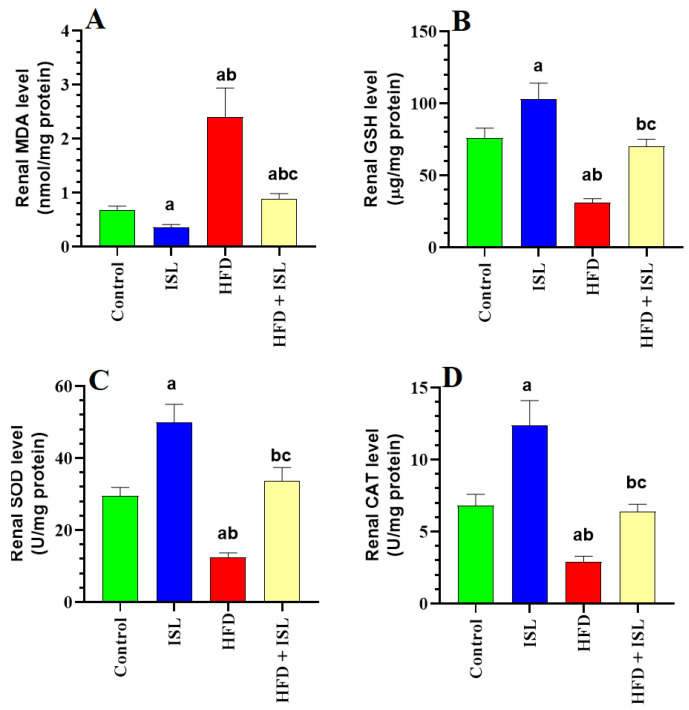
Levels of malondialdehyde (MDA) (**A**), glutathione (GSH) (**B**) and total levels of superoxide dismutase (SOD) (**C**) and catalase (**D**) in the kidneys of all groups of rats. Data are presented as means ± SD (*n* = 8/group). The values were significantly different at *p* < 0.05. ^a^: significantly different compared with control; ^b^: significantly different compared with ISL-treated rats (20 mg/kg); ^c^: significantly different compared with HFD-treated rats. MDA: malondialdehyde; SOD: superoxide dismutase; GSH: total glutathione; and CAT: catalase.

**Figure 2:**
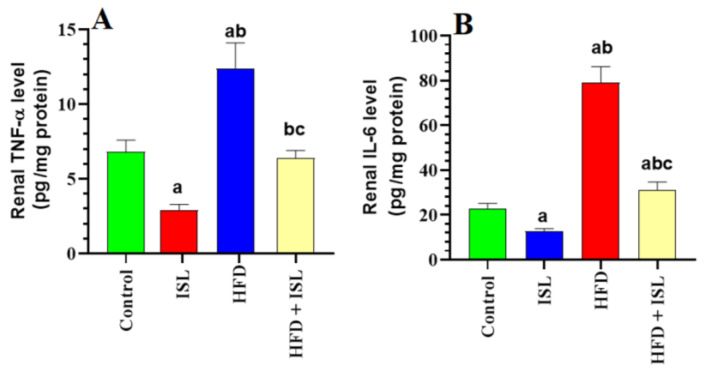
Levels of tumor necrosis factor-α (TNF-α) (**A**) and levels of interleukin-6 (IL-6) (**B**) in the kidneys of all groups of rats. Data are presented as means ± SD (*n* = 8/group). The values were significantly different at *p* < 0.05. ^a^: significantly different compared with control; ^b^: significantly different compared with ISL-treated rats (20 mg/kg); ^c^: significantly different compared with HFD-treated rats. TNF-α: tumor necrosis factor-α; IL-6: interleukin-6.

Levels of MDA, TNF-α, and IL-6 were significantly increased, whereas levels of GSH, SOD, and CAT were significantly reduced in the kidneys of the HFD-fed rats compared with the control rats (Figure 1A–D and Figure 2A,B). Levels of MDA, TNF-α, and IL-6 were significantly reduced, whereas levels of GSH, SOD, and CAT were significantly increased in the kidneys of both the ISL-treated and HFD + ISL-treated rats compared with the control and HFD-model rats, respectively (Figure 1A–D and Figure 2A,B).

### 3.5. ISL Downregulates MD-2, TLR4, and NF-κB and Reduces the Nuclear Translocation of NF-κB in the Kidneys of HFD-Fed Rats

**Figure 3:**
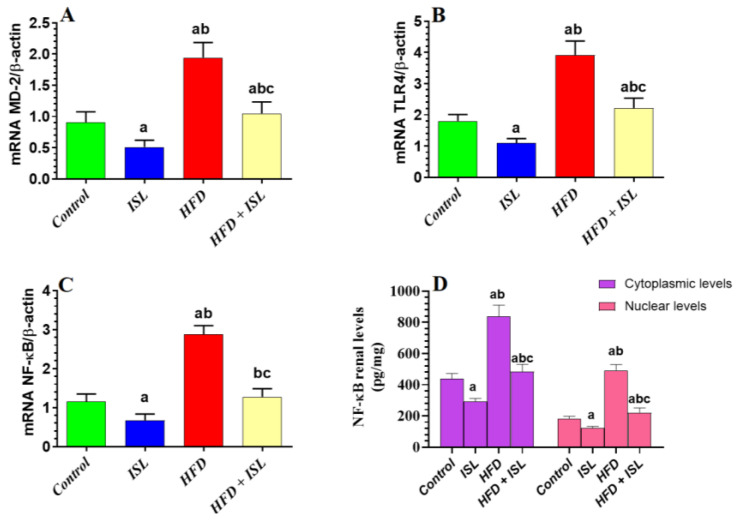
mRNA levels of MD-2 (**A**), TR4 (**B**), and NF-κB (**C**) as well as renal cytoplasmic and nuclear levels of NF-κB p65 (**D**) in all experimental groups of the study. Data are presented as means ± SD (*n* = 8/group). The values were significantly different at *p* < 0.05. ^a^: significantly different compared with control; ^b^: significantly different compared with ISL-treated rats (20 mg/kg); ^c^: significantly different compared with HFD-treated rats.

The mRNA levels of MD-2, TLR4, and NF-κB, as well as the total and nuclear levels of NF-κB p65, were significantly increased in the kidneys of the HFD-fed rats compared with the control rats (Figure 3A–D). mRNA levels of MD-2, TLR4, and NF-κB, as well as the total and nuclear levels of NF-κB p65, were significantly decreased in the kidneys of both the ISL-treated and HFD + ISL-treated rats compared with the control and HFD-model rats, respectively (Figure 3A–D).

### 3.6. ISL Preserves Kidney Structure and Reduces Tubular and Glomerular Damage in HFD-Fed Rats

**Figure 4:**
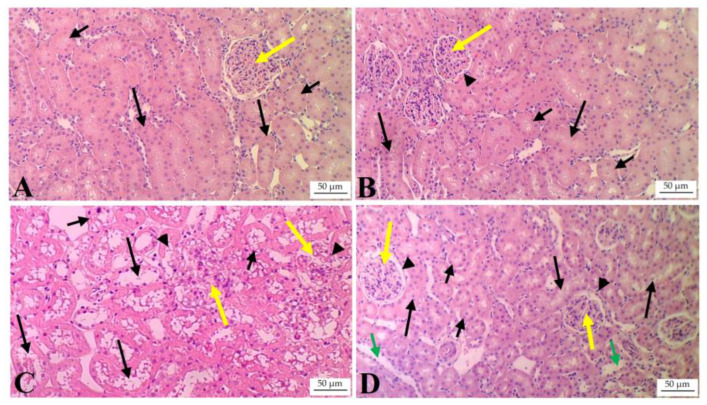
Histological images of the kidneys of all groups of rats. (**A**,**B**) were taken from control and ISL-treated rats and showed normal glomeruli (yellow arrow) with a normal-sized glomerular space and basement membrane (black arrowhead). Most proximal convoluted tubules (PCTs) (short black arrow) and distal convoluted tubules (DCTs) (long black arrow) were normal. (**C**) was taken from HFD-fed rats and showed abnormal glomerular shapes, with glomerular shrinkage; damaged, shrunk capillaries (yellow arrow); and damaged glomerular capsule (black arrowhead). The kidneys of this group of rats also showed severe degeneration and vacuolization in the PCTs (short black arrow) and DCTs (long black arrow). (**D**) was taken from HFD + ISL-treated rats and showed an almost normal glomerular shape (yellow arrow) and normally sized basement membrane (black arrowhead). The majority of the PCTs and DCTs had intact structures (short and long arrows, respectively). Tubular degeneration remained visible in some DCTs (short green arrow).

Kidneys of the control and ISL-treated rats showed normal histological features with intact glomeruli, glomerular space, and proximal and distal convoluted tubules (PCTs and DCTs, respectively) (Figure 4A,B). The glomeruli of the kidneys of HFD-fed rats were shrunk, and they showed damaged basement membranes (Figure 4C). There was also severe damage and vacuolization in the PCTs and DCTs. However, almost normal histological kidney structures, with very little damage in the glomeruli and tubules, were seen in the kidneys of HFD + ISL-treated rats with a few damaged areas in some of the DCTs (Figure 4D).

## 4. Discussion

**Figure 5:**
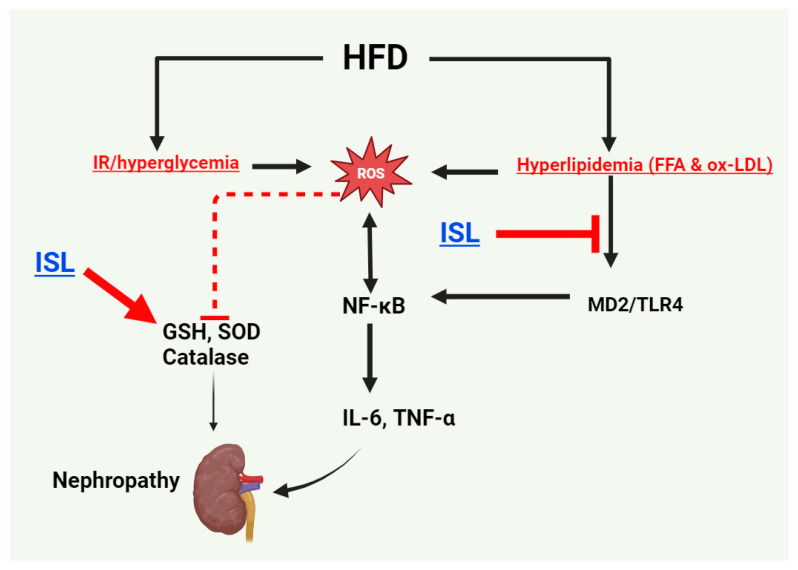
Graphical abstract demonstrating the precise nephroprotective effect of ISL against HFD-mediated nephropathy in HFD rats. ISL could suppress the generation of ROS by increasing the levels of antioxidants such as glutathione (GSH), superoxide dismutase (SOD), and catalase. In addition, ISL was able to suppress the activation of NF-kB by downregulating the MD2/TLR4, probably through its hypolipidemic and antioxidant effects, which decrease the generation of free fatty acids (FFAs) and the oxidized LDL cholesterols (ox-LDLs).

Previous studies have demonstrated the ability of ISL to prevent renal damage across various renal disorders [45,46]. This study extends this research by investigating ISL’s efficacy in alleviating HFD-mediated nephropathy. Our findings reveal significant potential for ISL to preserve kidney structure and function in an HFD animal model through several interconnected mechanisms: reducing IR and dyslipidemia, mitigating renal oxidative stress, enhancing the kidney’s endogenous antioxidants, and suppressing renal inflammation. However, ISL’s protective effects appear to stem primarily from its hypolipidemic properties and its capacity to alleviate renal inflammation by inhibiting the MD-2/TLR4/NF-κB signaling pathway. A graphical abstract demonstrating all these events is shown in Figure 5.

The Western diet is a significant contributor to CKD, largely due to its promotion of obesity and metabolic stress [8,50]. Evidence from numerous studies confirms the development of CKD in obese individuals and animals subjected to HFD [8,9]. Key indicators of CKD onset include albuminuria and low GFR, with novel biomarkers like KIM-1 and NGAL showing elevated levels in the urine and blood of CKD patients and animal models [51,52,53]. For instance, a positive correlation between high-fat intake and albuminuria was established in the Nurses’ Health Study, involving 3000 female participants [54]. Furthermore, both human and animal studies consistently report microalbuminuria, elevated albumin-to-creatinine ratios, and reduced GFR in subjects consuming HFD or a Western diet, independent of DM and hypertension [50]. Increased levels of renal and urinary vimentin, KIM-1, and NGAL have also been associated with nephrotoxicity in both human patients and Zucker rats on HFD [47,55,56,57]. Notably, glomerulosclerosis, severe proximal tubule damage, and interstitial fibrosis are major pathological changes observed in the kidneys of HFD-fed subjects [4,8,16]. Conversely, a healthier, low-fat diet is linked to a reduced risk of renal injuries [11].

In our study, HFD-fed rats showed significantly higher body weight and fat mass, including visceral and subcutaneous fat. These rats also exhibited severe tubular and glomerular damage, consistent with previous findings. Such pathological changes were associated with elevated creatinine clearance, urinary albumin, albumin-to-creatinine ratios, and levels of KIM-1 and NGAL, further confirming renal injury and CKD onset. Importantly, co-treatment with ISL ameliorated these markers, providing robust evidence for its nephroprotective effects against obesity and HFD-induced nephrotoxicity. We propose that ISL may alleviate obesity-related nephropathy by reducing body weight and fat deposition. Previous studies have similarly demonstrated the weight-lowering effects of ISL in comparable animal models [43]. However, we did not explore the precise mechanisms by which ISL influences body weight or food intake, as there were no significant changes in food consumption across the study groups. This aspect warrants further investigation in future studies.

Metabolic stress is a principal mechanism by which obesity induces nephrotoxicity [16]. Lipotoxicity, inflammation, and oxidative stress are significant contributors to the nephrotoxic effects of HFD and obesity [8]. Obesity is known to provoke renal damage through mechanisms involving IR and hyperglycemia, which exacerbate ROS production and oxidative stress [58,59,60]. Indeed, hyperglycemia leads to ROS overproduction in diabetic tissues, including the kidneys, via various pathways such as NADPH oxidase activation and advanced glycation end products [16,61]. The urinary marker 8-OHdG is frequently utilized to detect oxidative stress in the kidneys, with higher levels correlating with albuminuria severity in diabetic nephropathy [62,63]. Additionally, markers of lipid peroxidation, such as MDA and TBARS, alongside antioxidant levels, have been extensively used to evaluate oxidative stress in kidney tissues under various pathological conditions. In HFD-fed rats, MDA levels were significantly increased, while enzymatic and non-enzymatic antioxidants were depleted [16,57,64].

Consistent with these findings, the HFD-fed rats in our study developed a T2DM phenotype, as indicated by elevated plasma glucose and insulin levels and an increased HOMA-IR index. Renal tissues from these rats displayed significantly higher MDA levels, accompanied by elevated urinary 8-OHdG levels and decreased levels of GSH, CAT, and SOD. Conversely, co-treatment with ISL effectively prevented the development of IR, hyperglycemia, and hyperinsulinemia in the HFD-fed rats. Given that ISL did not impact glucose or insulin levels in the control group, we conclude that ISL exerts an insulin-sensitizing effect, likely due to its influence on body weight and adipose tissue synthesis. Previous studies have similarly highlighted ISL’s anti-hyperglycemic and insulin-sensitizing effects in HFD and diabetic models [43,65,66]. Furthermore, ISL reduced the MDA levels and enhanced the antioxidant levels in the kidneys of control rats, suggesting an antioxidant effect that is independent of hyperglycemia. Emerging evidence supports the notion that ISL’s therapeutic efficacy across various tissues is linked to its ability to scavenge ROS and upregulate antioxidants through the regulation of Nrf2 [43,44,67].

However, addressing hyperglycemia alone does not fully resolve renal damage in obese animal models [8,16]. The independent roles of dyslipidemia and renal lipotoxicity in CKD progression have become prominent areas of research [8]. FFAs serve as key energy substrates metabolized by tubular cells for energy homeostasis [68]. Thus, maintaining lipid homeostasis is crucial for normal renal reabsorption and excretory functions [8,68]. Lipid overload, particularly from saturated fats, is recognized as an independent nephrotoxic factor, promoting oxidative stress and inflammation through mechanisms that include mitochondrial damage and endoplasmic reticulum stress [8,69]. Lipid accumulation within the kidneys can result in vacuolar degeneration and epithelial cell detachment [8]. Biopsy samples from patients with diabetic nephropathy and HFD-fed animals have shown increased lipid deposition in the renal tubules [70,71,72,73,74,75]. Higher cholesterol levels have also been linked to increased mortality in CKD patients, even in the absence of renal inflammation and malnutrition [69,76]. In our study, dyslipidemia was evident in HFD-fed rats, as indicated by elevated serum levels of FFAs, cholesterol, triglycerides, LDL-c, and ox-LDL-c. These findings align with numerous previous studies examining HFD and Western diets’ effects on lipid profiles [16,69,72,77]. Notably, increased levels of TGs, cholesterol, and FFAs were also observed in the kidneys of HFD-fed rats, indicating renal lipotoxicity. Conversely, ISL’s ability to reduce serum and renal lipid levels in both the control and HFD-fed rats suggests a significant hypolipidemic effect. This indicates that ISL may offer novel therapeutic avenues for preventing and treating nephropathies by alleviating dyslipidemia and renal lipotoxicity. The lack of effects on food intake and other metabolic parameters in the control rats implies that ISL’s hypolipidemic effects are likely independent of these factors. Notably, the significant reduction in serum levels of ox-LDL in both the control and HFD-fed rats reinforces ISL’s antioxidant capacity, suggesting its potential to decrease renal uptake of FFAs and ox-LDL-c, although this requires further validation.

Accumulating evidence points to inflammation as a key mechanism in the pathogenesis of CKD [78,79]. Inflammation is widely believed to underlie the nephrotoxic effects of HFD in both rodents and humans [8,16]. The sustained activation of NF-κB is recognized as a central mechanism driving renal injury in CKD and in HFD-fed animals, leading to increased inflammation, oxidative stress, and apoptosis [18,28]. Suppression of NF-κB in obese models has demonstrated protective effects against nephropathy [20,21]. However, NF-κB activation is a complex process influenced by various factors [8]. Recent studies suggest that the MD2/TLR4 axis is a crucial contributor to HFD-induced nephropathy, directly stimulating NF-κB [8,21,31,32]. The inhibition of MD2 has been shown to preserve renal function and morphology, reducing oxidative damage in HFD-fed rodents [30,31].

In our study, HFD-fed rats exhibited significantly elevated renal mRNA levels of TLR4 and MD2, correlating with increased NF-κB activity and inflammatory markers like TNF-α and IL-6. These findings confirm that HFD induces a chronic inflammatory state in the kidneys. The elevated serum and renal levels of FFAs and ox-LDL-c observed in the HFD-fed rats may elucidate the increased expression of MD2 and TLR4. Saturated FFAs, particularly palmitic acid and ox-LDL, are known to upregulate these receptors, thereby activating NF-κB and inducing tissue inflammation [30,31,33,80]. Additionally, ROS can stimulate NF-κB directly [81], potentially exacerbating its activation. Remarkably, ISL was found to inhibit inflammation in the kidneys of HFD-fed rats, evidenced by reduced leukocyte infiltration and diminished TLR4 and MD2 expression, ultimately leading to a decrease in NF-κB activation and associated inflammatory cytokines. The finding that ISL treatment significantly attenuated renal inflammation suggests that ISL serves as a potent anti-inflammatory agent in the context of obesity-related nephropathy.

While our study provides significant insights into ISL’s nephroprotective effects against HFD-induced nephropathy, several limitations warrant consideration. Future studies should utilize more refined models to further elucidate ISL’s precise mechanisms of action, particularly regarding its effects on MD-2, and investigate the complex interplay among oxidative stress, lipid oxidation, and NF-κB activation.

## 5. Conclusions

Our research offers novel insights into ISL’s protective capabilities against HFD-induced nephrotoxicity through multifaceted mechanisms, including anti-obesity, antioxidant, hypolipidemic, and anti-inflammatory effects. The observed suppression of the MD-2/TLR4/NF-κB pathway highlights ISL’s therapeutic potential for obesity-related renal disorders. Further research is essential to unravel the underlying mechanisms and evaluate ISL’s applicability in clinical settings.

## 6. Study Limitations

This study has several limitations to consider. Firstly, it employed a specific animal model that may not fully reflect human physiology and the complexities of HFD-induced nephropathy. The intervention duration may also have been too short to capture the long-term effects and benefits of ISL. While various biomarkers were assessed, including additional markers of renal function could provide a more comprehensive picture. The precise mechanisms through which ISL affects body weight and fat deposition were not explored, leaving gaps in understanding its metabolic effects. The absence of placebo control makes it challenging to differentiate ISL’s effects from natural variations. Dietary factors beyond HFD were not controlled, potentially confounding outcomes. Histological analyses were limited, possibly overlooking other renal changes in the HFD-fed rats. The study also did not assess potential interactions between ISL and other medications or components in the HFD, which could influence its efficacy. Furthermore, a limitation of the current study is that ISL was co-administered with the HFD, which may not reflect an ideal therapeutic scenario. Future studies should consider administering ISL post-pathology development, as this could more closely mimic the potential clinical application of ISL as a treatment. Lastly, further research is needed to validate these findings across diverse populations and varying ISL doses for optimal therapeutic regimens.

## Figures and Tables

**Table 1:** Primer characteristics of the real-time PCR.

Gene	Primers (5′-3′)	Product Size
MD2	F: AAATCCCTATTTCAATTAGTTCTGAACC R: GAGTTGATATTGATGAACAGGTTGAAAT	130
NF-κB	F: GAGATTGTGCCAAGAGTGAC R: CTTGTCTTCCATGGTGGATG	134
TLR4	F: GATTGCTCAGACATGGCAGTTTC R: CTGCTAAGAAGGCGATACAATTCG	117
β-actin	F: GACCTCTATGCCAACACAGT R: CACCAATCCACACAGAGTAC	154

**Table 2:** Changes in final body weight and adiposity marker in all groups of rats.

	Parameter	Control	ISL	HFD	HFD + ISL
	Final body weight	454.5 ± 43.4	429 ± 51.6	584.4 ± 47.5 ^ab^	483.5 ± 51.3 ^abc^
Plasma	Glucose (mg/dL)	96.7 ± 7.61	103.5 ± 6.5	188.6 ± 15.4 ^ab^	137.3 ± 12.5 ^abc^
	Insulin (µIU/mL)	5.1 ± 0.49	4.8 ± 0.62	8.7 ± 0.93 ^ab^	6.3 ± 0.59 ^abc^
	HOMA-IR	1.27 ± 0.26	1.22 ± 0.37	3.92 ± 0.48 ^ab^	2.3 ± 0.31 ^abc^
WAT	Visceral fat (VF) (g)	8.1 ± 0.74	7.8 ± 0.93	13.2 ± 1.82 ^ab^	9.5 ± 0.79 ^abc^
	Subcutaneous fat (SF) (g)	18.6 ± 2.6	19.9 ± 1.9	34.5 ± 3.1 ^ab^	25.5 ± 2.9 ^abc^

Data are presented as means ± SD (*n* = 8/group). Values were significantly different at *p* < 0.05. ^a^: significantly different compared with control; ^b^: significantly different compared with ISL-treated rats (20 mg/kg); ^c^: significantly different compared with HFD-treated rats.

**Table 3:** Lipid profile in the serum and kidneys of all groups of rats.

	Parameter	Control	ISL	HFD	HFD + ISL
Serum	TGs (mg/dL)	74.3 ± 6.7	59.4 ± 5.8 ^a^	172 ± 16.6 ^ab^	89.4 ± 10.5 ^abc^
	CHOL (mg/dL)	86.7 ± 8.1	64.3 ± 5.4 ^a^	222.4 ± 18.9 ^ab^	104.3 ± 9.5 ^abc^
	LDL-c (mg/dL)	39.8 ± 4.7	31.2 ± 3.6 ^a^	127.4 ± 11.4 ^ab^	49.5 ± 4.3 ^abc^
	Ox-LDL-c (ng/mL)	26.5 ± 2.5	19.4 ± 1.5 ^a^	78.6 ± 6.4 ^ab^	37.5 ± 4.1 ^abc^
	FFAs (μmol/mg)	257.2 ± 22.1	189.3 ± 16.1 ^a^	598.3 ± 63.4 ^ab^	302 ± 26.5 ^abc^
Kidney	TGs (ng/g tissue)	137.3 ± 12.7	116.3 ± 12.3 ^a^	456.2 ± 39.4 ^ab^	219.4 ± 22.6 ^abc^
	CHOL (ng/g tissue)	27.6 ± 2.8	18.4 ± 1.4 ^a^	75.2 ± 6.1 ^ab^	39.7 ± 4.4 ^abc^
	FFAs (μmol/g tissue)	54.8 ± 4.9	38.3 ± 5.9 ^a^	163.7 ± 13.2 ^ab^	87.6 ± 5.1 ^abc^
	Ox-LDL (pg/g tissue)	122.5 ± 10.4	91.2 ± 7.9 ^a^	383.6 ± 27.6 ^ab^	178.5 ± 19.4 ^abc^

Data are presented as means ± SD (*n* = 8/group). The values were significantly different at *p* < 0.05. ^a^: significantly different compared with control; ^b^: significantly different compared with ISL-treated rats (20 mg/kg); ^c^: significantly different compared with HFD-treated rats.

**Table 4:** The renal function profile in all groups of rats.

	Parameter	Control	ISL	HFD	HFD + ISL
Serum	Kidney weight (g)	1.57 ± 0.16	1.49 ± 0.18	2.36 ± 0.24	1.63 ± 0.15 ^bc^
	Albumin (g/dL)	5.49 ± 0.44	5.77 ± 0.63	3.81 ± 0.39 ^ab^	5.39 ± 0.53 ^c^
	Urea (mg/dL)	6.42 ± 0.59	5.99 ± 0.51	34.5 ± 3.1 ^ab^	9.5 ± 1.5 ^abc^
	Creatinine (Cr) (μg/dL)	422.3 ± 40.4	466.8 ± 54.3	1221.2 ± 132.2 ^ab^	644.3 ± 53.4 ^abc^
Urine	Volume (ml/24 h)	13.5 ± 1.81	12.8 ± 1.76	8.5 ± 0.93 ^ab^	13.2 ± 1.45 ^abc^
	Albumin (Alb) (µg/dL)	87.4 ± 8.8	81.9 ± 7.6	227.5 ± 21.6 ^ab^	125.8 ± 12.1 ^abc^
	Creatinine (μg/dL)	322.5 ± 31.2	299 ± 33.2	125.6 ± 10.8 ^ab^	273 ± 22.7 ^abc^
	Urinary Alb/Cr ratio	0.24 ± 0.04	0.26 ± 0.05	2.18 ± 0.26 ^ab^	0.42 ± 0.06 ^abc^
	NGAL (ng/mL)	2.6 ± 0.38	2.7 ± 0.48	13.4 ± 1.6 ^ab^	3.7 ± 0.44 ^abc^
	8-OHdG (pg/mL)	320.2 ± 27.7	210.5 ± 18.6 ^a^	645.4 ± 43.9 ^ab^	382.5 ± 4.7 ^abc^
	KIM-1 (pg/mL)	133.2 ± 14.9	122.7 ± 12.6	292.4 ± 27.4 ^ab^	158.3 ± 13.2 ^abc^

Data are presented as means ± SD (*n* = 8/group). The values were significantly different at *p* < 0.05. ^a^: significantly different compared with control; ^b^: significantly different compared with ISL-treated rats (20 mg/kg); ^c^: significantly different compared with HFD-treated rats.

## Data Availability

The datasets used and analyzed during the current study are available from the corresponding author upon reasonable request.

## References

[1] Lv J.-C., Zhang L.-X. (2019). Prevalence and disease burden of chronic kidney disease. Ren. Fibros. Mech. Ther..

[2] Bikbov B., Purcell C.A., Levey A.S., Smith M., Abdoli A., Abebe M., Adebayo O.M., Afarideh M., Agarwal S.K., Agudelo-Botero M. (2020). Global, regional, and national burden of chronic kidney disease, 1990–2017: A systematic analysis for the Global Burden of Disease Study 2017. Lancet.

[3] Moreno J.A., Gomez-Guerrero C., Mas S., Sanz A.B., Lorenzo O., Ruiz-Ortega M., Opazo L., Mezzano S., Egido J. (2018). Targeting inflammation in diabetic nephropathy: A tale of hope. Expert Opin. Investig. Drugs.

[4] Rapa S.F., Di Iorio B.R., Campiglia P., Heidland A., Marzocco S. (2019). Inflammation and oxidative stress in chronic kidney disease—Potential therapeutic role of minerals, vitamins and plant-derived metabolites. Int. J. Mol. Sci..

[5] Wang V., Vilme H., Maciejewski M.L., Boulware L.E. (2016). The economic burden of chronic kidney disease and end-stage renal disease. Semin. Nephrol..

[6] Webster A.C., Nagler E.V., Morton R.L., Masson P. (2017). Chronic kidney disease. Lancet.

[7] Chen T.K., Knicely D.H., Grams M.E. (2019). Chronic kidney disease diagnosis and management: A review. JAMA.

[8] Chen S., Chen J., Li S., Guo F., Li A., Wu H., Chen J., Pan Q., Liao S., Liu H.-F. (2021). High-fat diet-induced renal proximal tubular inflammatory injury: Emerging risk factor of chronic kidney disease. Front. Physiol..

[9] Rao A.S., Camilleri M., Eckert D.J., Busciglio I., Burton D.D., Ryks M., Wong B.S., Lamsam J., Singh R., Zinsmeister A.R. (2011). Urine sugars for in vivo gut permeability: Validation and comparisons in irritable bowel syndrome-diarrhea and controls. Am. J. Physiol.-Gastrointest. Liver Physiol..

[10] Kramer H. (2019). Diet and chronic kidney disease. Adv. Nutr..

[11] Hariharan D., Vellanki K., Kramer H. (2015). The Western diet and chronic kidney disease. Curr. Hypertens. Rep..

[12] Lin J., Judd S., Le A., Ard J., Newsome B.B., Howard G., Warnock D.G., McClellan W. (2010). Associations of dietary fat with albuminuria and kidney dysfunction. Am. J. Clin. Nutr..

[13] He L.-Q., Wu X.-H., Huang Y.-Q., Zhang X.-Y., Shu L. (2021). Dietary patterns and chronic kidney disease risk: A systematic review and updated meta-analysis of observational studies. Nutr. J..

[14] Bach K.E., Kelly J.T., Palmer S.C., Khalesi S., Strippoli G.F., Campbell K.L. (2019). Healthy dietary patterns and incidence of CKD: A meta-analysis of cohort studies. Clin. J. Am. Soc. Nephrol..

[15] Thomas G., Sehgal A.R., Kashyap S.R., Srinivas T.R., Kirwan J.P., Navaneethan S.D. (2011). Metabolic syndrome and kidney disease: A systematic review and meta-analysis. Clin. J. Am. Soc. Nephrol. CJASN.

[16] Aldayel T.S. (2022). Apigenin attenuates high-fat diet-induced nephropathy in rats by hypoglycemic and hypolipidemic effects, and concomitant activation of the Nrf2/antioxidant axis. J. Funct. Foods.

[17] Imig J.D., Ryan M.J. (2013). Immune and inflammatory role in renal disease. Compr. Physiol..

[18] Zhang H., Sun S.-C. (2015). NF-κB in inflammation and renal diseases. Cell Biosci..

[19] Sanz A.B., Sanchez-Niño M.D., Ramos A.M., Moreno J.A., Santamaria B., Ruiz-Ortega M., Egido J., Ortiz A. (2010). NF-κB in renal inflammation. J. Am. Soc. Nephrol..

[20] Kim J.E., Lee M.H., Nam D.H., Song H.K., Kang Y.S., Lee J.E., Kim H.W., Cha J.J., Hyun Y.Y., Han S.Y. (2013). Celastrol, an NF-κB inhibitor, improves insulin resistance and attenuates renal injury in db/db mice. PLoS ONE.

[21] Fang Q., Deng L., Wang L., Zhang Y., Weng Q., Yin H., Pan Y., Tong C., Wang J., Liang G. (2015). Inhibition of Mitogen-Activated Protein Kinases/Nuclear Factor κB–Dependent Inflammation by a Novel Chalcone Protects the Kidney from High Fat Diet–Induced Injuries in Mice. J. Pharmacol. Exp. Ther..

[22] Sun Y., Peng R., Peng H., Liu H., Wen L., Wu T., Yi H., Li A., Zhang Z. (2016). miR-451 suppresses the NF-kappaB-mediated proinflammatory molecules expression through inhibiting LMP7 in diabetic nephropathy. Mol. Cell. Endocrinol..

[23] Hewage S.M., Prashar S., Debnath S.C., O K., Siow Y.L. (2020). Inhibition of inflammatory cytokine expression prevents high-fat diet-induced kidney injury: Role of lingonberry supplementation. Front. Med..

[24] Li S., Jia Y., Xue M., Hu F., Zheng Z., Zhang S., Ren S., Yang Y., Si Z., Wang L. (2020). Inhibiting Rab27a in renal tubular epithelial cells attenuates the inflammation of diabetic kidney disease through the miR-26a-5p/CHAC1/NF-kB pathway. Life Sci..

[25] Eleazu C., Suleiman J.B., Othman Z.A., Zakaria Z., Nna V.U., Hussain N.H.N., Mohamed M. (2022). Bee bread attenuates high fat diet induced renal pathology in obese rats via modulation of oxidative stress, downregulation of NF-kB mediated inflammation and Bax signalling. Arch. Physiol. Biochem..

[26] Knauf F., Brewer J.R., Flavell R.A. (2019). Immunity, microbiota and kidney disease. Nat. Rev. Nephrol..

[27] Xia H., Bao W., Shi S. (2017). Innate immune activity in glomerular podocytes. Front. Immunol..

[28] Song N., Thaiss F., Guo L. (2019). NFκB and kidney injury. Front. Immunol..

[29] Wang Y., Qian Y., Fang Q., Zhong P., Li W., Wang L., Fu W., Zhang Y., Xu Z., Li X. (2017). Saturated palmitic acid induces myocardial inflammatory injuries through direct binding to TLR4 accessory protein MD2. Nat. Commun..

[30] Fang Q., Wang L., Yang D., Chen X., Shan X., Zhang Y., Lum H., Wang J., Zhong P., Liang G. (2017). Blockade of myeloid differentiation protein 2 prevents obesity-induced inflammation and nephropathy. J. Cell. Mol. Med..

[31] Xu S., Luo W., Xu X., Qian Y., Xu Z., Yu W., Shan X., Guan X., Lum H., Zhou H. (2019). MD2 blockade prevents oxLDL-induced renal epithelial cell injury and protects against high-fat-diet-induced kidney dysfunction. J. Nutr. Biochem..

[32] Zhang Y., Chen H., Zhang W., Cai Y., Shan P., Wu D., Zhang B., Liu H., Khan Z.A., Liang G. (2020). Arachidonic acid inhibits inflammatory responses by binding to myeloid differentiation factor-2 (MD2) and preventing MD2/toll-like receptor 4 signaling activation. Biochim. Biophys. Acta (BBA)-Mol. Basis Dis..

[33] Song M., Meng L., Liu X., Yang Y. (2021). Feprazone prevents free fatty acid (FFA)-induced endothelial inflammation by mitigating the activation of the TLR4/MyD88/NF-κB pathway. ACS Omega.

[34] Zhang M., Huang L.-L., Teng C.-H., Wu F.-F., Ge L.-Y., Shi Y.-J., He Z.-L., Liu L., Jiang C.-J., Hou R.-N. (2018). Isoliquiritigenin provides protection and attenuates oxidative stress-induced injuries via the Nrf2-ARE signaling pathway after traumatic brain injury. Neurochem. Res..

[35] Peng F., Du Q., Peng C., Wang N., Tang H., Xie X., Shen J., Chen J. (2015). A review: The pharmacology of isoliquiritigenin. Phytother. Res..

[36] Ramalingam M., Kim H., Lee Y., Lee Y.-I. (2018). Phytochemical and pharmacological role of liquiritigenin and isoliquiritigenin from radix glycyrrhizae in human health and disease models. Front. Aging Neurosci..

[37] Al-Qahtani W.H., Alshammari G.M., Ajarem J.S., Al-Zahrani A.Y., Alzuwaydi A., Eid R., Yahya M.A. (2022). Isoliquiritigenin prevents Doxorubicin-induced hepatic damage in rats by upregulating and activating SIRT1. Biomed. Pharmacother..

[38] Wu Y., Chen X., Ge X., Xia H., Wang Y., Su S., Li W., Yang T., Wei M., Zhang H. (2016). Isoliquiritigenin prevents the progression of psoriasis-like symptoms by inhibiting NF-κB and proinflammatory cytokines. J. Mol. Med..

[39] Zou P., Ji H.-M., Zhao J.-W., Ding X.-M., Zhen Z.-G., Zhang X., Nie X.-Q., Xue L.-X. (2019). Protective effect of isoliquiritigenin against cerebral injury in septic mice via attenuation of NF-κB. Inflammopharmacology.

[40] Gao Y., Lv X., Yang H., Peng L., Ci X. (2020). Isoliquiritigenin exerts antioxidative and anti-inflammatory effects via activating the KEAP-1/Nrf2 pathway and inhibiting the NF-κB and NLRP3 pathways in carrageenan-induced pleurisy. Food Funct..

[41] Ye H., Yang X., Chen X., Shen L., Le R. (2020). Isoliquiritigenin protects against angiotensin II-induced fibrogenesis by inhibiting NF-κB/PPARγ inflammatory pathway in human Tenon’s capsule fibroblasts. Exp. Eye Res..

[42] Sun J., Zhang Q., Yang G., Li Y., Fu Y., Zheng Y., Jiang X. (2022). The licorice flavonoid isoliquiritigenin attenuates Mycobacterium tuberculosis-induced inflammation through Notch1/NF-κB and MAPK signaling pathways. J. Ethnopharmacol..

[43] Yahya M.A., Alshammari G.M., Osman M.A., Al-Harbi L.N., Yagoub A.E.A., AlSedairy S.A. (2022). Liquorice root extract and isoliquiritigenin attenuate high-fat diet-induced hepatic steatosis and damage in rats by regulating AMPK. Arch. Physiol. Biochem..

[44] Yahya M.A., Alshammari G.M., Osman M.A., Al-Harbi L.N., Yagoub A.E.A., AlSedairy S.A. (2022). Isoliquiritigenin attenuates high-fat diet-induced intestinal damage by suppressing inflammation and oxidative stress and through activating Nrf2. J. Funct. Foods.

[45] Tang Y., Wang C., Wang Y., Zhang J., Wang F., Li L., Meng X., Li G., Li Y., Wang L. (2018). Isoliquiritigenin attenuates LPS-induced AKI by suppression of inflammation involving NF-κB pathway. Am. J. Transl. Res..

[46] Liao Y., Tan R.-Z., Li J.-C., Liu T.-T., Zhong X., Yan Y., Yang J.-K., Lin X., Fan J.-M., Wang L. (2020). Isoliquiritigenin attenuates UUO-induced renal inflammation and fibrosis by inhibiting Mincle/Syk/NF-Kappa B signaling pathway. Drug Des. Devel. Ther..

[47] Kim K.S., Yang H.Y., Song H., Kang Y.R., Kwon J., An J., Son J.Y., Kwack S.J., Kim Y.-M., Bae O.-N. (2017). Identification of a sensitive urinary biomarker, selenium-binding protein 1, for early detection of acute kidney injury. J. Toxicol. Environ. Health A.

[48] Folch J., Lees M., Sloane Stanley G.H. (1957). A simple method for the isolation and purification of total lipids from animal tissues. J. Biol. Chem..

[49] Bazzano A., Wolfe C., Zylowska L., Wang S., Schuster E., Barrett C., Lehrer D. (2015). Mindfulness based stress reduction (MBSR) for parents and caregivers of individuals with developmental disabilities: A community-based approach. J. Child Fam. Stud..

[50] Wahba I.M., Mak R.H. (2007). Obesity and obesity-initiated metabolic syndrome: Mechanistic links to chronic kidney disease. Clin. J. Am. Soc. Nephrol..

[51] Wang J.-G., Staessen J.A., Barlassina C., Fagard R., Kuznetsova T., Struijker-Boudier H.A., Zagato L., Citterio L., Messaggio E., Bianchi G. (2002). Association between hypertension and variation in the α-and β-adducin genes in a white population. Kidney Int..

[52] Kohl K., Herzog E., Dickneite G., Pestel S. (2020). Evaluation of urinary biomarkers for early detection of acute kidney injury in a rat nephropathy model. J. Pharmacol. Toxicol. Methods.

[53] Zhou H., Cui J., Lu Y., Sun J., Liu J. (2021). Meta-analysis of the diagnostic value of serum, plasma and urine neutrophil gelatinase-associated lipocalin for the detection of acute kidney injury in patients with sepsis. Exp. Ther. Med..

[54] Knight E.L., Stampfer M.J., Hankinson S.E., Spiegelman D., Curhan G.C. (2003). The impact of protein intake on renal function decline in women with normal renal function or mild renal insufficiency. Ann. Intern. Med..

[55] Vaidya V.S., Ferguson M.A., Bonventre J.V. (2008). Biomarkers of acute kidney injury. Annu. Rev. Pharmacol. Toxicol..

[56] Fiseha T. (2015). Urinary biomarkers for early diabetic nephropathy in type 2 diabetic patients. Biomark. Res..

[57] Sun Y., Ge X., Li X., He J., Wei X., Du J., Sun J., Li X., Xun Z., Liu W. (2020). High-fat diet promotes renal injury by inducing oxidative stress and mitochondrial dysfunction. Cell Death Dis..

[58] Sharma K. (2009). The link between obesity and albuminuria: Adiponectin and podocyte dysfunction. Kidney Int..

[59] Saravanan S., Pari L. (2016). Protective effect of thymol on high fat diet induced diabetic nephropathy in C57BL/6J mice. Chem.-Biol. Interact..

[60] Kovesdy C.P., Furth S., Zoccali C., Committee W.K.D.S. (2017). Obesity and kidney disease: Hidden consequences of the epidemic. Physiol. Int..

[61] Kashihara N., Haruna Y., K Kondeti V., S Kanwar Y. (2010). Oxidative stress in diabetic nephropathy. Curr. Med. Chem..

[62] Kundu A., Richa S., Dey P., Kim K.S., Son J.Y., Kim H.R., Lee S.-Y., Lee B.-H., Lee K.Y., Kacew S. (2020). Protective effect of EX-527 against high-fat diet-induced diabetic nephropathy in Zucker rats. Toxicol. Appl. Pharmacol..

[63] Xu G., Yao Q., Weng Q., Su B., Zhang X., Xiong J. (2004). Study of urinary 8-hydroxydeoxyguanosine as a biomarker of oxidative DNA damage in diabetic nephropathy patients. J. Pharm. Biomed. Anal..

[64] Rangel Silvares R., Nunes Goulart da Silva Pereira E., Eduardo Ilaquita Flores E., Lino Rodrigues K., Ribeiro Silva A., Gonçalves-de-Albuquerque C.F., Daliry A. (2019). High-fat diet-induced kidney alterations in rats with metabolic syndrome: Endothelial dysfunction and decreased antioxidant defense. Diabetes Metab. Syndr. Obes. Targets Ther..

[65] Gaur R., Yadav K.S., Verma R.K., Yadav N.P., Bhakuni R.S. (2014). In vivo anti-diabetic activity of derivatives of isoliquiritigenin and liquiritigenin. Phytomedicine.

[66] Yang L., Jiang Y., Zhang Z., Hou J., Tian S., Liu Y. (2020). The anti-diabetic activity of licorice, a widely used Chinese herb. J. Ethnopharmacol..

[67] Zhang W., Wang G., Zhou S. (2018). Protective effects of isoliquiritigenin on LPS-induced acute lung injury by activating PPAR-γ. Inflammation.

[68] Gewin L.S. (2021). Sugar or Fat? Renal tubular metabolism reviewed in health and disease. Nutrients.

[69] Kochan Z., Szupryczynska N., Malgorzewicz S., Karbowska J. (2021). Dietary lipids and dyslipidemia in chronic kidney disease. Nutrients.

[70] Bobulescu I.A. (2010). Renal lipid metabolism and lipotoxicity. Curr. Opin. Nephrol. Hypertens..

[71] Kiss E., Kränzlin B., Wagenblaβ K., Bonrouhi M., Thiery J., Gröne E., Nordström V., Teupser D., Gretz N., Malle E. (2013). Lipid droplet accumulation is associated with an increase in hyperglycemia-induced renal damage: Prevention by liver X receptors. Am. J. Pathol..

[72] Herman-Edelstein M., Scherzer P., Tobar A., Levi M., Gafter U. (2014). Altered renal lipid metabolism and renal lipid accumulation in human diabetic nephropathy. J. Lipid Res..

[73] Khan S., Jawdeh B.G.A., Goel M., Schilling W.P., Parker M.D., Puchowicz M.A., Yadav S.P., Harris R.C., El-Meanawy A., Hoshi M. (2014). Lipotoxic disruption of NHE1 interaction with PI (4, 5) P2 expedites proximal tubule apoptosis. J. Clin. Investig..

[74] Yamamoto T., Takabatake Y., Takahashi A., Kimura T., Namba T., Matsuda J., Minami S., Kaimori J.-y., Matsui I., Matsusaka T. (2017). High-fat diet–induced lysosomal dysfunction and impaired autophagic flux contribute to lipotoxicity in the kidney. J. Am. Soc. Nephrol. JASN.

[75] Yao R.-Q., Ren C., Xia Z.-F., Yao Y.-M. (2021). Organelle-specific autophagy in inflammatory diseases: A potential therapeutic target underlying the quality control of multiple organelles. Autophagy.

[76] Liu Y., Coresh J., Eustace J.A., Longenecker J.C., Jaar B., Fink N.E., Tracy R.P., Powe N.R., Klag M.J. (2004). Association between cholesterol level and mortality in dialysis patients: Role of inflammation and malnutrition. JAMA.

[77] Rahman M., Yang W., Akkina S., Alper A., Anderson A.H., Appel L.J., He J., Raj D.S., Schelling J., Strauss L. (2014). Relation of serum lipids and lipoproteins with progression of CKD: The CRIC study. Clin. J. Am. Soc. Nephrol. CJASN.

[78] Silverstein D.M. (2009). Inflammation in chronic kidney disease: Role in the progression of renal and cardiovascular disease. Pediatr. Nephrol..

[79] Akchurin O.M., Kaskel F. (2015). Update on inflammation in chronic kidney disease. Blood Purif..

[80] Rocha D., Caldas A., Oliveira L., Bressan J., Hermsdorff H. (2016). Saturated fatty acids trigger TLR4-mediated inflammatory response. Atherosclerosis.

[81] Morgan M.J., Liu Z.-G. (2010). Reactive oxygen species in TNFα-induced signaling and cell death. Mol. Cells.

